# Retina pathology as a target for biomarkers for Alzheimer's disease: Current status, ophthalmopathological background, challenges, and future directions

**DOI:** 10.1002/alz.13529

**Published:** 2023-11-02

**Authors:** Jessica Alber, Femke Bouwman, Jurre den Haan, Robert A. Rissman, Lies De Groef, Maya Koronyo‐Hamaoui, Imre Lengyel, Dietmar Rudolf Thal, the Alzheimer's Association ISTAART “The Eye as a Biomarker for AD” Professional Interest Area

**Affiliations:** ^1^ George and Anne Ryan Institute for Neuroscience, Department of Biomedical and Pharmaceutical Sciences University of Rhode Island Kingston Rhode Island USA; ^2^ Butler Hospital Memory & Aging Program Providence Rhode Island USA; ^3^ Amsterdam UMC, location VUmc Alzheimer Center, Department of Neurology Amsterdam The Netherlands; ^4^ Alzheimer's Therapeutic Research Institute Keck School of Medicine of the University of Southern California San Diego California USA; ^5^ Cellular Communication and Neurodegeneration Research Group, Animal Physiology and Neurobiology Division, Department of Biology Leuven Brain Institute KU Leuven Leuven Belgium; ^6^ Departments of Neurosurgery, Neurology, and Biomedical Sciences Maxine Dunitz Neurosurgical Research Institute, Cedars‐Sinai Medical Center Los Angeles California USA; ^7^ The Wellcome‐Wolfson Institute for Experimental Medicine, School of Medicine, Dentistry and Biomedical Science Queen's University Belfast Belfast UK; ^8^ Laboratory of Neuropathology Department of Imaging and Pathology, and Leuven Brain Institute, KU Leuven Leuven Belgium; ^9^ Department of Pathology UZ Leuven Leuven Belgium

**Keywords:** Alzheimer's disease, amyloid pathology, imaging, recruitment of clinical trials, retina, tau pathology

## Abstract

There is emerging evidence that amyloid beta protein (Aβ) and tau‐related lesions in the retina are associated with Alzheimer's disease (AD). Aβ and hyperphosphorylated (p)‐tau deposits have been described in the retina and were associated with small amyloid spots visualized by in vivo imaging techniques as well as degeneration of the retina. These changes correlate with brain amyloid deposition as determined by histological quantification, positron emission tomography (PET) or clinical diagnosis of AD. However, the literature is not coherent on these histopathological and in vivo imaging findings. One important reason for this is the variability in the methods and the interpretation of findings across different studies. In this perspective, we indicate the critical methodological deviations among different groups and suggest a roadmap moving forward on how to harmonize (i) histopathologic examination of retinal tissue; (ii) in vivo imaging among different methods, devices, and interpretation algorithms; and (iii) inclusion/exclusion criteria for studies aiming at retinal biomarker validation.

## 1 INTRODUCTION

In the United States, the prevalence of Alzheimer's disease (AD) is expected to more than double from an estimated 6.7 to 14 million by 2060, and at least 55 million people are currently living with AD or other dementias worldwide.^1^ In AD, amyloid beta peptide (Aβ) and abnormally phosphorylated tau (p‐tau) protein accumulations precede synaptic dysfunction, neurodegeneration, cognitive decline, and dementia, respectively.^2^, ^3^, ^4^, ^5^, ^6^, ^7^ Interestingly, biomarkers indicating Aβ deposition are currently more sensitive than those for tau and neurodegeneration.^8^, ^9^ Accurate diagnosis and timely intervention at the preclinical/asymptomatic stage of AD are core aims of drug development. Early intervention is posited to offer the best chance of therapeutic success. The development and validation of several disease‐specific biomarkers – Aβ or tau positron emission tomography (PET), cerebrospinal fluid (CSF) assays, and magnetic resonance imaging (MRI) – led to the amyloid/tau/neurodegeneration (AT[N]) framework.^10^ AT(N) emphasizes the characterization of AD based on biomarker tests and pathology rather than clinical presentation. Existing reference standard biomarkers (used to determine AT[N] status and detect AD risk) are limited by invasiveness, required administration by specialist teams, lack of accessibility, and high cost. A critical need exists to develop minimally invasive, scalable, cost‐effective, and accessible AD risk screening and/or disease‐monitoring biomarkers. In light of forthcoming, efficient disease‐modifying treatments,^11^, ^12^ disease‐monitoring biomarkers and the assessment of amyloid‐related imaging abnormalities (ARIA) become even more important.

Recently, plasma biomarkers have emerged as a viable alternative to identify symptomatic AD; however, validation and use of plasma biomarkers to identify preclinical/asymptomatic AD is still under investigation, with some data showing that plasma biomarkers alone may lack sensitivity and specificity compared to reference standard biomarkers (CSF, amyloid PET) in preclinical AD.^9^, ^13^, ^14^ In addition, plasma biomarkers lack the spatial information on the site of neuronal and vascular injury and can be influenced by interference from peripheral tissue, as well as inflammatory and metabolic processes.^15^

The retina is a promising target for developing minimally invasive, scalable, cost‐effective, and accessible AD risk screening or disease‐monitoring markers. Its shared embryologic origin and cellular composition with the brain,^16^, ^17^, ^18^ its direct connection with several brain regions, its accessibility by point‐of‐care clinicians using standard ophthalmological techniques, and data showing that visual changes manifest in the earliest stages of AD^19^, ^20^, ^21^, ^22^, ^23^, ^24^ render the retina an ideal AD risk biomarker target. Several retinal imaging techniques have been developed to determine AD‐associated lesions.^25^ Thus, considerable evidence indicates that there is neurodegeneration in the symptomatic and preclinical AD retina that can be measured by optical coherence tomography (OCT) – a non‐invasive retinal imaging technique widely applied to diagnose and monitor retinopathies.^25^, ^26^, ^27^ For example, retinal thinning had been measured in autosomal dominant presenilin 1 (*PSEN1*) mutation carriers, even in minimally symptomatic cases, indicating the diagnostic potential of the retina.^28^, ^29^ Similarly, retinal vasculature and perfusion changes are well established,^26^ with tortuosity as also observed by retinal fundus imaging,^30^ vascular Aβ_40_ and Aβ_42_ deposits, perivascular microglial activation, and tight junction loss in histological examinations of retinas from mild cognitive impairment (MCI) and AD patients^31^, ^32^, ^33^, ^34^ as well as in animal models of AD.^35^, ^36^ Retinal arterial Aβ_40_ burden correlates with cerebral amyloid angiopathy severity in these patients.^32^ Blood flow heterogeneity has also been described in the retinas of *PSEN1* and amyloid precursor protein gene (*APP*) mutant AD cases.^37^ These markers are not specific to AD and could represent neurodegeneration in general. However, they could be of significant value as part of a suite of retinal AD biomarkers. The histopathology literature examining the animal and human retina for AD‐specific proteinopathies (Aβ and p‐tau) is controversial to a certain extent. Several cross‐sectional studies have shown Aβ‐positive material in the retina of AD patients in vivo^34^, ^38^, ^39^, ^40^, ^41^, ^42^ and ex vivo^34^, ^43^, ^44^, ^45^, ^46^, ^47^, ^48^, ^49^, as well as p‐tau.^34^, ^44^, ^45^, ^50^ Numerous independent studies have revealed Aβ deposits and p‐tau accumulations in the retina of rodent models of AD.^43^, ^51^, ^52^, ^53^, ^54^, ^55^ However, a few studies have shown a lack of signal of amyloidosis or tauopathy in the retina in at least a subset of AD patients^56^ or atypical morphologies and staining pattern (very small deposits, corpora amylacea‐related staining) of anti‐Aβ‐stained material.^44^, ^57^ Tau pathology remains an underexplored factor in the AD retina. Nevertheless, animal and human work shows the presence of p‐tau in the retina.^44^, ^50^, ^54^ Given the biochemical pattern of retinal p‐tau in western blot analysis, a primary retinal tauopathy (PRET) has been discussed as a potential precursor for retinal AD p‐tau pathology similar to primary age‐related tauopathy (PART) in the brain.^57^ Furthermore, the spectral signatures of Aβ_42_ and pS396tau in the human AD retina have been described recently and represent an attractive target for a future in vivo label‐free method.^58^

Very recently, proteome signatures of the human AD retinas and brains were reported pointing to elevated inflammatory markers and cellular components (microgliosis and astrogliosis), defective microglial function, oxidative stress, and mitochondrial markers, along with markers of neurodegeneration, especially of the photoreceptors in the retina of AD patients.^59^ Similar processes were observed in proteomics studies from human AD brains.^60^, ^61^

This work promotes a link between retinal and cerebral changes in AD by indicating parallels between AD pathologies in the brain and retina from MCI and AD patients, including preclinical AD cases.^32^, ^34^, ^43^, ^44^, ^45^, ^46^, ^50^, ^55^, ^59^, ^60^, ^61^, ^62^, ^63^ Multiple clinical studies using retinal imaging modalities support a relationship between the retina and the brain in AD,^34^, ^38^, ^39^, ^40^, ^41^, ^42^, ^64^, ^65^, ^66^, ^67^, ^68^, ^69^, ^70^, ^71^, ^72^, ^73^ as well as studies using animal models for AD pathology.^43^, ^51^, ^55^, ^74^, ^75^, ^76^, ^77^, ^78^, ^79^, ^80^ Hence, while many studies have found structural, proteomic, cellular, or vascular changes in the retina of AD and/or preclinical AD patients and healthy controls, a few others have failed to find significant group differences. There are several possible reasons for this, not the least of which is that retinal imaging equipment and/or techniques, methods of analysis, and study populations vary widely across studies. Standardization of the respective approaches for recruitment, in vivo imaging, histopathology, and image analysis in these studies is required to use retinal imaging as a viable screening or disease‐monitoring biomarker for AD.

From this perspective, we identify key challenges for retinal imaging and its current and future position as a biomarker in symptomatic and preclinical AD for risk estimation and disease monitoring. Specifically, we look at three challenges that must be addressed to advance ocular biomarkers through the next step of the pipeline: (1) lack of a standardized histopathological approach to examining and interpreting Aβ and p‐tau pathologies in the retina as a gold standard for retinal biomarker validation, (2) lack of standardized data collection and image processing across imaging modalities and devices, and (3) lack of cogent inclusion/exclusion criteria for participants in AD biomarker development clinical studies. We will review these areas in what follows and provide recommendations to address each challenge as the field progresses.

**RESEARCH IN CONTEXT**

1. **Systematic review**: A literature review using online databases was conducted. We report the current knowledge on retinal Alzheimer's disease (AD) pathology, its visualization with imaging techniques, and its validation in clinical trials. We traced problems regarding comparability of different studies in the literature. Appropriate literature was cited.
2. **Interpretation**: There is a need for harmonizing standards for (1) the assessment and description of retinal neurodegenerative pathologies, (2) the use of distinct retinal imaging applications for determining neurodegenerative features in the retina, and (3) validation in clinical trials.
3. **Future directions**: The goal is to clarify the extent to which retinal imaging can be used to detect AD neurodegenerative changes. If so, we need to find out which retinal imaging techniques can be used for screening patients for their risk of developing AD and which may be better suited for disease monitoring, especially in the context of therapeutic trials.

## 2 KEY CHALLENGES FOR THE RETINA AS A BIOMARKER FOR AD

### 2.1 Lack of standardized histopathological approach for examining AD‐related pathology in the retina

The main challenges for the pathological validation of the retinal imaging findings are (i) the reproducibility of the pathological examination by different groups obtained with different stainings and biochemical techniques, (ii) the link of retinal Aβ and p‐tau pathologies with AD and/or other pathological conditions of the brain, (iii) the pathogenetic meaning of retinal pathologies, for example, early stage versus end stage phenomena, spreading routes, biochemical maturation of protein aggregate composition over time,^81^, ^82^ and (iv) the determination of the impact of other non‐AD pathologies on retinal integrity, for example, frontotemporal lobar degeneration (FTLD‐tau) or TDP‐43 proteinopathies for different diagnostic purposes.^50^, ^83^

Histopathological analysis of retina samples from AD patients has led to discordant findings.^43^, ^44^, ^50^, ^56^, ^84^ Some groups have been able to show extracellular Aβ‐positive material in the retina of AD patients in flatmounts (flatmounts are whole retina pieces that are stained as a whole and mounted in a manner to see the entire surface of the retina) and cross sections of the paraffin‐embedded retina.^43^, ^45^, ^48^, ^49^, ^84^ In this context, flatmount specimens appeared to have a higher sensitivity for the detection of amyloid material and better comparability with retinal imaging techniques but do not offer convincing information on the laminar distribution of Aβ deposits throughout the layers of the retina unless confocal imaging techniques are used.^34^, ^43^, ^48^ Other groups did not report the presence of histopathologically detectable extracellular retinal amyloid plaque‐like structures,^44^, ^47^, ^56^ although these groups reported intraneuronal Aβ positivity,^47^ corpora amylacea‐like extracellular bodies in both AD and controls,^44^ or dot‐like very small Aβ deposits.^45^ Moreover, the presence of Aβ oligomers has also been discussed based on positive staining results with conformation‐dependent antibodies.^59^, ^85^ Therefore, harmonization of pathological methods and their context of use, staining protocols, and interpretation of findings are required. This will be critical to validate the presence of “Aβ deposits” seen with novel ophthalmological imaging methods. Non‐specific staining and cross reactivity with other proteins need to be excluded for the antibodies used to stain Aβ material in the retina, and flatmount results will need to be confirmed in cross sections from paraffin‐embedded tissue. Pioneering studies^34^ will need to be confirmed. Moreover, when confirmed, the retinal Aβ deposits should be integrated into the terminology of Aβ plaque types reported in the brain by clearly mentioning their specific features, such as a presumably smaller size compared to cerebral plaques.^86^ Notably, transmission electron microscopy (TEM) analysis has also confirmed the existence of Aβ_42_ deposits and classical fibrils and protofibrils in the AD retina, which were identical in ultrastructure to their counterparts in the brain.^31^, ^34^, ^59^

Several groups have shown p‐tau pathology in the retina,^34^, ^44^, ^87^ while one was unable to demonstrate retinal tau.^56^ In AD cases, even argyrophilic neurofibrillary tangles (NFTs) have been described.^34^ However, in non‐AD controls, various levels of p‐tau pathology were observed,^44^ and a potential link to glaucoma was reported.^87^ Accordingly, there remains a lack of understanding on whether p‐tau lesions in preclinical AD cases can be found in the retina or whether non‐AD retinal tauopathic changes occur independently of AD as considered in glaucoma cases.^87^ An experimental mouse model for chronic traumatic injury showed that chronic exposure to head trauma led to a retinal tauopathy,^88^ further arguing in favor of a non‐specific, reactive nature of retinal tau pathology occurring under various pathological conditions. Accordingly, as with retinal Aβ deposits, it is essential to clarify the similarities and differences between retinal p‐tau pathology and cerebral tauopathies, including AD and non‐AD tauopathies. Recently published data on retinal p‐tau biochemistry revealed significant differences between the retinal p‐tau pattern and that of brain lysates from AD and PART brain, suggesting that retinal p‐tau pathology may represent a distinct PRET as a potential prerequisite for retinal AD tauopathy.^57^ Accordingly, questions arise about the link of retinal Aβ and p‐tau pathology with that in the brain: Is there a specific disease stage when the retina becomes involved in AD tauopathy or in Aβ deposition? Is there propagation of Aβ and/or p‐tau from the retina to the brain or vice versa?

In addition to Aβ and p‐tau, other pathologies can contribute to the development of dementia in the AD brain, such as limbic‐predominant age‐related TDP‐43 encephalopathy (LATE), Lewy body disease (LBD), cerebrovascular pathology, and so forth. It will be important to clarify whether these pathologies also play a role in the retina. LBD pathology has already been well documented to affect the retina.^89^, ^90^ Moreover, the first reports on TDP‐43 pathology in the retina are available.^83^, ^91^ Accordingly, there is a need to clarify whether such retinal copathologies may interfere with a potentially diagnostic value of retinal Aβ and p‐tau for the diagnosis of AD.

Thus, it is essential to address all these challenges to put the use of retinal imaging biomarkers for AD on solid ground and to validate imaging techniques based on pathological findings. Moreover, the harmonization of biochemical/biophysical methods for the analysis of proteins will be required in addition to that for histopathological examination, especially when investigating oligomers.^92^

### 2.2 Lack of standardized data collection and image processing across in vivo imaging modalities and devices

The techniques used for in vivo retinal image collection depend on the biomarker being studied. OCT is used to measure the thickness/volume of retinal layers.^69^, ^70^, ^93^ Vessel density, foveal avascular zone size, and perfusion density can be measured using OCT‐angiography (OCTA) and color fundus images.^73^ Hyperspectral imaging, fluorescence lifetime imaging ophthalmoscopy (FLIO), and confocal scanning laser ophthalmoscopy with fundus (auto)fluorescence are used to examine molecular and anatomical changes in retinal proteinopathies, including amyloid deposits.^29^, ^39^, ^40^, ^43^, ^58^, ^94^ Metabolic changes in terms of blood oxidation are measured using retinal oximetry.^95^ The use of ultrawide‐field color and autofluorescence imaging showed that extending observations of retinal morphology to the peripheral retina should also be considered.^96^, ^97^, ^98^ Which of these imaging techniques can be used for AD risk identification as a screening tool and which are better suited for monitoring disease progression and following treatment success remains undefined and requires standardization as well.

There is a critical need for agreement on basic standards for acquiring and analyzing retinal images, dependent on the imaging technique, to allow data sharing across research groups, similar to determining retinal degeneration in optic neuritis and multiple sclerosis by OCT.^99^ This would offer the opportunity to compare imaging data across study cohorts and eventually create a widely available comparative reference database, similar to the Alzheimer's disease neuroimaging initiative (ADNI) database for AD biomarker development.^100^ This would significantly improve how we acquire and interpret retinal imaging markers for Alzheimer's disease diagnosis, assessment, and disease monitoring.

One of the main challenges is that although instrument manufacturers produce high‐quality imaging systems that are in clinical and research use worldwide, the technical specifications may vary across manufacturers and even across different devices of the same manufacturer. This causes differences in resolution, repeated measurements, and post‐processing algorithms when working in a network using multiple imaging devices. Moreover, even when it comes to the same manufacturer, software updates and changes in resolution can result in subtle differences in segmentation algorithms, which can create noise in the data.

Developing a standardized minimum use data set for retinal biomarker development will require cooperation between academic research groups and the industry to develop algorithms for data conversion among different instruments, as previously done for diabetic retinopathy^101^ and for amyloid PET with the centiloid scale.^102^ The aim should be to begin with widely available clinical tools that can be deployed by point‐of‐care clinicians for the development and validation of retinal biomarkers. Spectral domain OCT (SD‐OCT) and confocal scanning laser ophthalmoscopy (cSLO) are commonly available in clinical settings and are a good starting point for creating a minimum data set for retinal imaging biomarker development in AD.

An additional challenge is that several investigators in academia and industry are developing novel experimental technologies and engineering advances that may supersede the technologies we currently rely on for retinal thickness measurements, retinal protein changes, and retinal angiography. Machine learning algorithms are being trained to detect AD‐related changes and validated on retinal photographs.^103^, ^104^ In this context, it will be essential to develop a mechanistic understanding of retinal changes throughout the AD spectrum before applying machine learning algorithms to detect, diagnose, or monitor AD‐related changes to ensure the validity of the respective techniques.

Thus, the aim should be to reach a consensus on reproducible methodological standards and standard operating procedures (SOPs) that can be used across laboratories and ophthalmologists to collect data, cross‐validate findings, and accelerate the development of sensitive and specific retinal biomarkers for the detection of AD‐related pathologic changes.

### 2.3 Harmonization of inclusion/exclusion criteria for participants in retinal AD biomarker development studies

One of the major sources of non‐comparable data across studies is variability in inclusion/exclusion criteria for AD patients and healthy controls. To develop robust guidelines for inclusion/exclusion of participants and biomarker standards across disciplines, communication and collaboration between AD and retinal researchers are essential. Although memory clinic‐based research cohorts are well aware of the importance of biomarker‐confirmed diagnosis, ophthalmology researchers are often unaware of these biomarkers and clinical criteria used to diagnose preclinical/prodromal/clinical AD in the Alzheimer's research field. On the other hand, memory clinic researchers lack experience in retinal imaging and are often unaware of ophthalmological and/or other clinical conditions that may affect adequate retinal measurements. An example is that a standardized ophthalmological investigation is often lacking, and other eye diseases, such as diabetic retinopathy, macular degeneration, glaucoma, and so forth, that could influence the imaging results are often not recorded. This leads to different between‐group comparisons and makes aggregating data across research cohorts difficult. Harmonization of enrollment of participants in retinal AD biomarker studies is a desirable step to move retinal biomarkers from the development to the validation phase of the biomarker pipeline. Therefore, it will be necessary to (i) develop a list of ophthalmological diseases that should exclude patients from enrollment in retinal AD imaging research and (ii) define the requirements of biomarker information across clinical AD stages for all patients enrolled in retinal AD imaging research. Questionnaires for retinal AD biomarker studies, based on standard optometry/ophthalmology practice, still need to be developed for screening for ophthalmologic disorders. In addition, standardized criteria, including biomarkers for the enrollment of preclinical/asymptomatic AD, MCI, and symptomatic stages of the AD continuum in studies examining novel biomarkers, are not yet established. The AT(N) framework offers a framework for the determination of biomarker‐positive “asymptomatic AD” cases as it changes the diagnosis of AD from a clinically defined symptomatic disease toward a biological definition that includes asymptomatic, biomarker‐positive AD cases.^10^ But even here a large spectrum of biomarkers can be used,^10^ whereas reproducible standards (eg, specific gold standard biomarkers for Aβ and p‐tau, or histopathological validation in end‐of‐life studies) are required for the validation of novel biomarkers.

The intersection of ophthalmological and AD diagnostics in research offers the opportunity to combine different recruitment strategies, such as memory clinic‐, optometry/ophthalmology clinic‐, and community‐based cohorts. This facilitates collaborations between every research field (ie, ophthalmological, neurodegenerative and epidemiological research) to advance the development of a retinal biomarker.

## 3 FUTURE PERSPECTIVES FOR THE DEVELOPMENT OF VALID RETINAL IN VIVO IMAGING BIOMARKERS

After identifying the main challenges for using the retina as a biomarker for AD with a focus on risk estimation and disease monitoring, we want to discuss options on how to address these challenges. To point out a roadmap based on the priorities of the respective tasks, we will describe potential short‐term solutions, long‐term goals, and possible strategies to reach them (Table 1).

**TABLE 1:** Short‐term and long‐term goals for establishing the retina as a biomarker for Alzheimer's disease.

(i) Short‐term goals	
*Goals*	*Action points*
Harmonization of research parameters and readouts for the eye as a biomarker in AD for pathological analysis	‐ Standardized protocols for tissue isolation, preservation, embedding, and processing in predefined anatomical regions ‐ Standardized protocols for immunohistochemistry of Aβ, p‐tau, and so forth ‐ Standardized protocols for reading and interpretation ‐ Integration of retinal neurodegenerative lesions in respective terminologies used for brain lesions of respective proteins
Harmonization of research parameters and readouts for the eye as a biomarker in AD for imaging	‐ Recommendations for choice of imaging technology to determine distinct retinal measures ‐ Comparability of measurements among different imaging devices; development of centiloid‐like scales for distinct imaging techniques ‐ Standardized interpretation protocols
Harmonization of selection and stratification criteria for integration of patients/samples in studies focusing on retina as biomarker for AD	‐ Definition of inclusion and exclusion criteria for studies using retinal biomarkers ‐ Definition of proper stratification criteria ‐ Definition of standard biomarkers for validation

(ii) Long‐term goals	
*Goals*	*Action points*
Understand pathobiological meaning of neurodegenerative processes occurring in the retina and their link to AD and other neurodegenerative disorders or normal aging	‐ Transgenic animal research for understanding the role of retinal Aβ and p‐tau pathology in the context of brain disease ‐ Analysis of seeding and propagation routes, including retina ‐ Determination of relevant Aβ‐ and p‐tau‐related tissue injury (degeneration, inflammation) and copathologies (vasculopathy, mitochondrial dysfunction, and other proteinopathies) in the human retina
Determine clinical and pathobiological relevance of retinal changes observed with novel retinal imaging techniques	‐ Animal experiment comparing imaging techniques with histopathology ‐ Human end‐of‐life studies or preoperative imaging studies comparing imaging results with histopathology

Abbreviation: AD, Alzheimer's disease.

Given our analysis of the challenges, several steps can be taken in the short term:

(i) harmonization of research parameters and readouts for the eye as a biomarker in AD for pathological analysis,
(ii) harmonization of research parameters and readouts for the eye as a biomarker in AD for in vivo imaging, and
(iii) harmonization of selection and stratification criteria for integrating patients/samples in studies focusing on the retina as a biomarker for AD.

In the long term, it will be essential to

(iv) understand the pathobiological meaning of the neurodegenerative processes occurring in the retina and their link to AD and other neurodegenerative disorders or normal aging and
(v) determine the clinical and pathobiological relevance of retinal changes observed with novel retinal imaging techniques.

### 3.1 Short‐term solutions required for retinal biomarker development

#### 3.1.1 Harmonization of research parameters and readouts for the retina as a biomarker in AD for pathological analysis

The techniques and experimental settings in studies focusing on retinal pathologies in AD vary strikingly.^34^, ^43^, ^44^, ^47^, ^48^, ^56^ For example, pathological studies use many different antibodies/staining techniques to determine Aβ deposits and p‐tau pathology, as well as different fixation, processing, and staining protocols. Regarding the use of fixation methods and antibodies, one can rely on the work of Brain Net Europe for harmonizing staining and fixation techniques to determine amyloid deposits and NFTs in the human AD brain.^105^, ^106^ However, preparation and fixation strategies vary significantly for the retina. On the one hand, the entire retina or parts of the retina can be stained as a flatmount/wholemount specimen. This technique allows an extensive regional sampling and will find even single Aβ deposits in the retina. A convincing layer‐specific analysis would either require confocal imaging of the specimen and costaining with markers making it possible to visualize the anatomy of the retina, or subsequent paraffin embedding of cross sections to document the identified Aβ deposits with this technique. Paraffin embedding and cross sectioning of the retina allow excellent visualization of the retinal layers but cover less retina surface than flatmount samples. Thus, some Aβ deposits may be missed.

Recommendations are required for the interpretation of ophthalmopathological lesions in the retina using formalin‐fixed, paraffin‐embedded (FFPE) tissue sections and flatmounts. The interpretation of the respective samples needs to take into account methodological limitations of each of the techniques. Given the sparsity of donated *post mortem* retina samples, residual tissue from biopsy/resection specimens taken primarily for other clinical indications is only available as FFPE tissue blocks and should not be excluded from research on neurodegenerative retina disorders. Another aspect that requires harmonization is the sample size and the location of retinal samples. Optimally, all areas of the retina should be systematically covered, that is, the posterior pole, including the macula region, the optic disc, and the peripheral retina. This can be performed in a standardized manner when the entire eyeball is available. In the case of evisceration biopsies and small retinal pieces received from biobanks, good coverage and/or orientation of the whole retina cannot be guaranteed. Moreover, a standardized screening scheme for relevant copathologies, such as macular degeneration, diabetic retinopathy, and so forth, would help make retina studies more comparable.

Thus, a basic set of samples needs to be defined and, if possible, include the use of whole eyeball resection specimens that are taken in surgical procedures to remove eye tumors. Here, the tumor diagnosis, staging, and determination of the status of the resection margins have priority, and only residual material can be used for research, provided informed consent is obtained. Such a basic setting can be supplemented by an additional sampling of retina parts under pure research conditions for flatmount preparations, if possible, as well as for biochemical preparations. Thus, additional sampling will presumably be restricted to retinas obtained in specific autopsy recruitment programs, best from cases also receiving brain autopsy. Furthermore, confirmation of the histological and immunocytochemical results with biochemical and/or functional methods will complete the interpretation of the retinal involvement in AD pathology.

It is also essential to agree on how to report and interpret pathological findings in retinal samples. Harmonized baseline standards that allow comparison of results are needed and should include a comprehensive strategy to assess Aβ, p‐tau, and other neurodegenerative features of the retina in a standardized manner.

In summary, we can define the following list of harmonization tasks for improving the comparability of pathological research on neurodegenerative disorders in the retina (see also action points in Table 1):

- Definition of biopsy/tissue types and their potential use in research: (a) paraffin‐embedded autopsy eye samples, (b) retinal flatmount samples from autopsy eyes, (c) frozen retina tissue, (d) residual samples from surgical eyeball resections, and (e) evisceration samples and their specific applications for research and diagnostics;
- Definition of retinal parts (central vs peripheral) to be analyzed (eg, assessment of Aβ deposits and p‐tau pathology);
- Definition of fixation and processing methods and indication under research and diagnostic conditions;
- Definition of antibodies and staining protocols suitable for staining retinal pathologies;
- Harmonized reading and reporting of neurodegenerative lesions, for example, Aβ deposits, tau pathology, and so forth;
- Harmonized reading and reporting of copathologies and reactive/inflammatory parameters;
- Establishment of protocols allowing quantitative assessments based on sample types (i) a to d (see earlier items);
- Determination of pathological meaning of retinal pathologies for underlying neurodegenerative disorders such as AD.

#### 3.1.2 Harmonization of research parameters and readouts for the retina as a biomarker in AD for retinal imaging

To harmonize the production and interpretation of retinal imaging data, we must begin with ophthalmological imaging techniques and devices that are widely available in clinics. It is essential to determine which imaging methods can be used for a distinct purpose, for example, detecting retinal amyloid deposits or thickness of the retina, and whether a given method provides information about early disease stages or can be better used for disease monitoring. Standard image acquisition techniques will be required for each retinal imaging modality. Guidance from existing retinal grading centers will help to design grading protocols for the different imaging modalities.^101^, ^107^ Separate recommendations are needed for the use, interpretation, and processing of both widely available and accessible methods such as cSLO and OCT and other available/emerging imaging methods such as hyperspectral imaging, fundus (auto)fluorescence imaging, OCTA, and FLIO.^29^ Equally important are the standardization guidelines for interpreting, scoring, and reporting each type of retinal imaging technique. We need to ensure that we are uniformly identifying and reporting AD‐related changes, whether structural, angiographic, metabolic, or protein‐related. Minimum standards for reporting should be established for each modality to accelerate cross‐validation across studies. In doing so, we will also need to define the context of use for all proposed retinal biomarkers. Appreciating that changes may be dynamic, longitudinal investigations will be essential for this purpose, along with relating retinal changes to visual functional measures.

Second, standardized data acquisition procedures in image acquisition, segmentation algorithms, post‐processing, interpretation/scoring, and reporting will be essential to develop and validate robust retinal biomarkers of AD. Frequent software upgrades and advances in imaging technologies that include upgrades or modifications to signal analyses complicate this issue. Moreover, sharing comparable images in a central platform is required to expedite biomarker validation. Agreement between academics, policymakers, and industry is required, allowing open access to raw imaging data in order to achieve this goal. Harmonized standards for imaging data, analogous to centiloids for amyloid PET, will be essential for the comparison of the generated data. By establishing such standards, we will derive methods for comparisons of images across different device manufacturers.

Reporting for imaging must be standardized to reliably compare data across sites and institutions for understanding in vivo retinal biomarker acquisition in AD and other neurodegenerative diseases.

Accordingly, we need standardized guidelines for interpreting, scoring, and reporting each type of retinal imaging result. We need to ensure that we are uniformly identifying and reporting identical changes with all devices and that they are AD‐related, regardless of structural, angiographic, metabolic, or protein‐related. Minimum standards for reporting should be established for each modality to accelerate cross‐validation across studies.

Finally, AD does not occur in a vacuum. Most older adults will have neurological, metabolic, and/or cardiovascular comorbidities. Establishing standardized methods to identify and separate these comorbidities from AD‐related changes using retinal imaging techniques will be essential for the long‐term monitoring of AD patients.

In summary, to receive comparable and valid imaging results, we need to take the following steps (see also action points in Table 1):

- Define the context of use for each imaging method.
- Establish relative measures that allow comparisons between different instruments for a given imaging technique (eg, different cameras for OCT, OCTA, cSLO, fundus autofluorescence, and so forth).
- Establish an ADNI‐like data‐sharing platform for retinal images/data sharing and transparency.
- Standardize operating procedures to carry out retinal imaging with each technique.
- Develop standardized guidelines for interpreting, scoring, and reporting all imaging techniques.
- Establish a protocol for assessing copathologies in the eye/retina and algorithms to correct them if necessary.

#### 3.1.3 Harmonization of selection and stratification criteria for integration of patients/samples in studies focusing on the retina as a biomarker for AD

When recruiting individuals for studies on retinal imaging, it will be essential to carefully screen potential participants not only for AD changes with established biomarkers, such as amyloid PET, but also for copathologies in both the brain and retina/eye. For example, a high number of vascular lesions in the brain can have an impact on the cognitive performance of an individual. Thus, dementia in such a patient may not be caused by AD alone. In fact, up to 60% of AD cases show evidence of concomitant non‐AD‐type pathologies.^108^, ^109^ Accordingly, the measurement of only amyloid pathology via retinal imaging will not be sufficient to cover all factors that cause dementia in a given patient. Therefore, cases with multiple pathologies need to be either excluded from studies determining the value of retinal imaging for predicting AD or considered a separate group in the analysis. An alternative is to control the statistical analysis in general for the respective copathologies as control variables. On the other hand, severe cataracts can interfere with the performance of retinal imaging devices. Accordingly, such patients may need to be excluded for technical reasons. Other retina pathologies, such as macular degeneration, retinal detachment, and so forth, may also impact the imaging results of the retina, and it will be necessary to consider such cases as a separate group or to exclude them from the respective studies.

Accordingly, it is essential to provide recommendations based on the agreement of researchers involved in such studies. Clear guidelines for stratification or exclusion will be essential to generate valid and reproducible results in clinical studies.

In summary, we can define the following list of harmonization tasks for selecting and stratifying patients/samples (see also action points in Table 1):

- Definition of explicit inclusion and exclusion criteria for studies that rely on the eye as a biomarker;
- Establishing a protocol for the ophthalmological examination to assess all relevant copathologies;
- Definition of stratification guidelines, for example, copathologies and mandatory control parameters.

### 3.2 Long‐term goals for retinal biomarker development

#### 3.2.1 Understanding of pathobiological meaning of neurodegenerative pathologies in the retina and their link to AD, Lewy body disease, and other neurodegenerative disorders

To understand the biological meaning of neurodegenerative retinal lesions, there is, in our opinion, a need for more systematic histo(patho)logical and biochemical analysis (including proteomics/transcriptomics and so forth) of human retina samples, not only comparing AD versus controls but also in a large number of retinas of different age groups with and without other retinal lesions such as glaucoma, macular degeneration and so forth. This analysis of human retinas needs to be supplemented by experimental data from models of the respective pathologies. It will teach us the meaning of Aβ and p‐tau pathologies in the retina and their association with AD and various non‐AD neurodegenerative conditions that may affect the retina. Since neurodegenerative diseases in the retina are not restricted to AD, it will also be essential to study retinas from patients with other tauopathies of the FTLD‐tau spectrum, LBD (which has already been documented in the retina^90^), TDP‐43 proteinopathies,^83^ and chronic traumatic encephalopathy, as well as rarer neurodegenerative disorders. It will also be important to determine the correlation of the neurodegenerative lesions with the loss of retinal neurons – both the extent of neuronal loss and the subtypes of affected neurons.

This general understanding of the role of neurodegenerative changes in the retina and of retinal cellular integrity and its relationship to distinct neurodegenerative disorders of the brain will be important for defining the role of visual symptoms and their relationship with underlying neuropathology. For exmple, sleep disturbances and dysregulated circadian rhythm reflect the loss of melanopsin‐positive retinal ganglion cells in AD patients.^33^

Moreover, neuron‐to‐neuron spreading of p‐tau^110^, ^111^ and Aβ^112^, ^113^ has been considered an important mechanism for disease propagation in AD. Whether similar mechanisms apply to retinal p‐tau and Aβ pathology is unknown. The prerequisite for answering this question is the clarification at which stages in the neuropathological expansion of Aβ and p‐tau pathology in the brain (Aβ phase, according to Thal et al.^113^ and Braak NFT stage^114^) the retina becomes involved. Animal models will be indispensable for studying predicted spreading routes, in addition to comprehensive anatomopathological studies of the retinas of AD patients in different stages of the disease requiring the availability of eye–brain donations.

Addressing these challenges to the pathogenetic and biological understanding of neurodegenerative changes in the retina will inevitably result in determination of the context of use for retinal AD. Possible action points are provided in Table 1.

#### 3.2.2 Clinical and pathobiological relevance of retinal changes observed with novel retinal imaging techniques

Pathological or molecular validation of findings obtained by novel retinal imaging techniques is essential to interpret findings correctly. Such validation requires animal models, in the first instance, which are already widely used in the field.^35^, ^43^, ^89^ However, in the end, confirmation in patients is essential. For this, end‐of‐life or preoperative imaging studies can be an option to confirm retinal imaging results with pathological findings in the retina. Similar validation of retinal imaging devices may be possible by including elderly patients who undergo enucleation of the eye for other purposes and in whom the devices can be tested shortly before surgery. Larger in vivo biomarker datasets will also be available to validate retinal biomarkers using established AD biomarkers such as CSF p‐tau, CSF Aβ, amyloid and tau PET, and blood‐based biomarkers for Aβ and p‐tau. Possible action points are provided in Table 1.

## 4 CONCLUSION

We aim to address the challenges of establishing retinal imaging as a biomarker for AD, especially for its risk and for disease monitoring, for example, under therapy. We should be able to establish the value of emerging retinal imaging techniques for diagnosing AD risk and disease progression and its differentiation from other neurodegenerative pathologies in the retina. In our opinion, the parallel application of harmonization measures and a better pathobiological understanding of the role of the retina in neurodegenerative disorders will help to position retinal biomarkers in the diagnostic workup, including disease monitoring and/or screening of neurodegenerative diseases and retina disorders. If these general measures prove successful in achieving validation and harmonization of retinal imaging, they may also help biomarker validation in AD in general and/or improve inclusion/exclusion criteria and/or stratification criteria for clinical trials. Comparable standards for such trials would be an enormous advantage. They will hopefully contribute to the diagnosis of AD and its accompanied copathologies in a personalized manner as a basis for future personalized therapy for neurodegenerative disorders.

## AUTHOR CONTRIBUTIONS

Conceptualization: Dietmar Rudolf Thal, Jessica Alber, Femke Bouwman, Robert A. Rissman, and Jurre den Haan. First manuscript draft and manuscript finalization: Dietmar Rudolf Thal and Jessica Alber. Supplementary manuscript drafting and review: Femke Bouwman, Robert A. Rissman, Maya Koronyo‐Hamaoui, Jurre den Haan, Lies De Groef, and Imre Lengyel. Consensus meeting: Dietmar Rudolf Thal, Jessica Alber, Maya Koronyo‐Hamaoui, Femke Bouwman, Lies De Groef, and Robert A. Rissman.

Table: Dietmar Rudolf Thal.

Review was offered to all members of the ISTAART – The eye as a biomarker for AD professional interest area (PIA).

## CONFLICT OF INTEREST STATEMENT

FB received a speaker honorarium from Roche and Biogen and has research collaborations with Optina Diagnostics (Canada) and Optos (UK). MKH is a cofounding member and consultant of NeuroVision Imaging, Inc. (Sacramento, CA, USA). IL has a research collaboration with OPTOS Plc and Hoffman La Roche. DRT received speaker honorarium or travel reimbursement from Biogen (USA) and UCB (Brussels, Belgium) and collaborated with Novartis Pharma AG (Basel, Switzerland), Probiodrug (Halle [Saale], Germany), GE‐Healthcare (Amersham, UK), and Janssen Pharmaceutical Companies (Beerse, Belgium). No authors reported relevant disclosures relevant to this work. Author disclosures are available in the supporting information.

## CONSENT STATEMENT

This article does not include any original data on human individuals. Therefore, consent of study participants is not applicable for this perspective article.

## Supporting information

Supporting Information

## 1

List of collaborators from Alzheimer's Association ISTAART “The Eye as a Biomarker for AD” Professional Interest Area (PIA):

Feedback was received from the following PIA members (in alphabetic order):

- *Melanie C. W. Campbell*—Physics and Astronomy, School of Optometry and Vision Science and Systems Design Engineering, University of Waterloo, 200 University Ave W, Waterloo, Ontario, N2L3G1, Canada
- *Vivek Kumar Gupta*—Macquarie Medical School, Faculty of Medicine, Health and Human Sciences, Macquarie University, 75 Talavera Road, Macquarie University, Sydney, NSW, 2109, Australia
- *Jeroen J.M. Hoozemans*—Pathology, Amsterdam Neuroscience, Amsterdam UMC—Vrije Universiteit Amsterdam, De Boelelaan 1117, 1081 HV Amsterdam, the Netherlands
- *Giulia Quattrini—*Laboratory of Alzheimer's Neuroimaging and Epidemiology (LANE), IRCCS Istituto Centro San Giovanni di Dio Fatebenefratelli, Via Pilastroni 4, 25125, Brescia, Italy
- *Urmimala Raychaudhuri*—University of California, Irvine, 419 S Circle View Dr (ISEB), 2500 La Spada Lab, Irvine, CA 92617, USA
- *Usman Saeed*—1. Institute of Medical Science, Faculty of Medicine, University of Toronto, Toronto, ON, Canada and 2. Sunnybrook Research Institute, Sunnybrook Health Sciences Centre, 2075 Bayview Avenue, Room M6 166, Toronto, ON, M4N 3M5, Canada.
- *Jean‐Philippe Sylvestre*—Optina Diagnostics, 8200 Boulevard Décarie, Suite 220, Montreal, Quebec, H4P 2P5, Canada.
- *Alice S. Tang*—Bakar Computational Health Sciences Institute, University of California San Francisco (UCSF), 490 Illinois St, Floor 2, Box 2933, San Francisco, CA 94143, USA

PIA administrators from ISTAART who reviewed the manuscript are as follows:

*Rebecca M. Edelmayer*—ISTAART, Alzheimer's Association, 225 N. Michigan Ave., 17th Floor, Chicago, IL 60601, USA
*Jodi R. Titiner*—ISTAART, Alzheimer's Association, 225 N. Michigan Ave., 17th Floor, Chicago, IL 60601, USA

Other members of the PIA who did not provide separate feedback are not considered collaborators. The views and opinions expressed by the authors in this publication represent those of the authors and do not necessarily reflect those of the PIA membership, ISTAART, or the Alzheimer's Association. For more information about “The Eye as a Biomarker for Alzheimer's Disease” PIA please visit the PIA webpage: https://istaart.alz.org/groups/home/73.

## References

[1] Association As . 2023 Alzheimer's disease facts and figures. Alzheimers Dement. 2023;19:1598‐1695. 36918389 10.1002/alz.13016

[2] Braak H , Thal DR , Ghebremedhin E , Del Tredici K . Stages of the pathologic process in Alzheimer disease: age categories from 1 to 100 years. J Neuropathol Exp Neurol. 2011;70:960‐969. 22002422 10.1097/NEN.0b013e318232a379

[3] Thal DR , Arendt T , Waldmann G , et al. Progression of neurofibrillary changes and PHF‐tau in end‐stage Alzheimer's disease is different from plaque and cortical microglial pathology. Neurobiol Aging. 1998;19:517‐525. 10192210 10.1016/s0197-4580(98)00090-6

[4] Thal DR , Griffin WS , Braak H . Parenchymal and vascular Abeta‐deposition and its effects on the degeneration of neurons and cognition in Alzheimer's disease. J Cell Mol Med. 2008;12:1848‐1862. 18624777 10.1111/j.1582-4934.2008.00411.x PMC4506155

[5] Thal DR , Holzer M , Rüb U , et al. Alzheimer‐related tau‐pathology in the perforant path target zone and in the hippocampal stratum oriens and radiatum correlates with onset and degree of dementia. Exp Neurol. 2000;163:98‐110. 10785448 10.1006/exnr.2000.7380

[6] Gomez‐Isla T , Price JL , McKeel Jr DW , Morris JC , Growdon JH , Hyman BT . Profound loss of layer II entorhinal cortex neurons occurs in very mild Alzheimer's disease. J Neurosci. 1996;16:4491‐4500. 8699259 10.1523/JNEUROSCI.16-14-04491.1996 PMC6578866

[7] Masliah E , Mallory M , Hansen L , DeTeresa R , Alford M , Terry R . Synaptic and neuritic alterations during the progression of Alzheimer's disease. Neurosci Lett. 1994;174:67‐72. 7970158 10.1016/0304-3940(94)90121-x

[8] Jack Jr CR , Holtzman DM . Biomarker modeling of Alzheimer's disease. Neuron. 2013;80:1347‐1358. 24360540 10.1016/j.neuron.2013.12.003 PMC3928967

[9] Therriault J , Pascoal TA , Lussier FZ , et al. Biomarker modeling of Alzheimer's disease using PET‐based braak staging. Nature Aging. 2022;2(6):526‐535. 37118445 10.1038/s43587-022-00204-0 PMC10154209

[10] Jack Jr CR , Bennett DA , Blennow K , et al. NIA‐AA research framework: toward a biological definition of Alzheimer's disease. Alzheimers Dement. 2018;14:535‐562. 29653606 10.1016/j.jalz.2018.02.018 PMC5958625

[11] Sims JR , Zimmer JA , Evans CD , et al. Donanemab in early symptomatic Alzheimer disease: the TRAILBLAZER‐ALZ 2 randomized clinical trial. JAMA. 2023;330(6):512‐527. 37459141 10.1001/jama.2023.13239 PMC10352931

[12] Lalli G , Schott JM , Hardy J , De Strooper B . Aducanumab: a new phase in therapeutic development for Alzheimer's disease? EMBO Mol Med. 2021;13:e14781. 34338436 10.15252/emmm.202114781 PMC8350893

[13] Thijssen EH , La Joie R , Strom A , et al. Plasma phosphorylated tau 217 and phosphorylated tau 181 as biomarkers in Alzheimer's disease and frontotemporal lobar degeneration: a retrospective diagnostic performance study. Lancet Neurol. 2021;20:739‐752. 34418401 10.1016/S1474-4422(21)00214-3 PMC8711249

[14] Barthelemy NR , Horie K , Sato C , Bateman RJ . Blood plasma phosphorylated‐tau isoforms track CNS change in Alzheimer's disease. J Exp Med. 2020;217. 10.1084/jem.20200861 PMC7596823 32725127

[15] Huber H , Ashton NJ , Schieren A , et al. Levels of Alzheimer's disease blood biomarkers are altered after food intake‐A pilot intervention study in healthy adults. Alzheimers Dement. 2023. doi:10.1002/alz.13163 37243891

[16] Erskine L , Herrera E . Connecting the retina to the brain. ASN Neuro. 2014;6. 10.1177/1759091414562107 PMC4720220 25504540

[17] Crair MC , Mason CA . Reconnecting eye to brain. J Neurosci. 2016;36:10707‐10722. 27798125 10.1523/JNEUROSCI.1711-16.2016 PMC5083002

[18] Patton N , Aslam T , Macgillivray T , Pattie A , Deary IJ , Dhillon B . Retinal vascular image analysis as a potential screening tool for cerebrovascular disease: a rationale based on homology between cerebral and retinal microvasculatures. J Anat. 2005;206:319‐348. 15817102 10.1111/j.1469-7580.2005.00395.x PMC1571489

[19] Risacher SL , WuDunn D , Tallman EF , et al. Visual contrast sensitivity is associated with the presence of cerebral amyloid and tau deposition. Brain Commun. 2020;2:fcaa019. 32309804 10.1093/braincomms/fcaa019 PMC7151662

[20] Salamone G , Di Lorenzo C , Mosti S , et al. Color discrimination performance in patients with Alzheimer's disease. Dement Geriatr Cogn Disord. 2009;27:501‐507. 19451717 10.1159/000218366

[21] Rizzo M , Anderson SW , Dawson J , Nawrot M . Vision and cognition in Alzheimer's disease. Neuropsychologia. 2000;38:1157‐1169. 10838150 10.1016/s0028-3932(00)00023-3

[22] Javitt DC , Martinez A , Sehatpour P , et al. Disruption of early visual processing in amyloid‐positive healthy individuals and mild cognitive impairment. Alzheimers Res Ther. 2023;15:42. 36855162 10.1186/s13195-023-01189-7 PMC9972790

[23] Sadun AA , Borchert M , DeVita E , Hinton DR , Bassi CJ . Assessment of visual impairment in patients with Alzheimer's disease. Am J Ophthalmol. 1987;104:113‐120. 3618708 10.1016/0002-9394(87)90001-8

[24] Trick GL , Trick LR , Morris P , Wolf M . Visual field loss in senile dementia of the Alzheimer's type. Neurology. 1995;45:68‐74. 7824139 10.1212/wnl.45.1.68

[25] Snyder PJ , Alber J , Alt C , et al. Retinal imaging in Alzheimer's and neurodegenerative diseases. Alzheimers Dement. 2021;17:103‐111. 33090722 10.1002/alz.12179 PMC8062064

[26] Shi H , Koronyo Y , Rentsendorj A , et al. Retinal vasculopathy in Alzheimer's disease. Front Neurosci. 2021;15:731614. 34630020 10.3389/fnins.2021.731614 PMC8493243

[27] Alber J , Goldfarb D , Thompson LI , et al. Developing retinal biomarkers for the earliest stages of Alzheimer's disease: what we know, what we don't, and how to move forward. Alzheimers Dement. 2020;16:229‐243. 31914225 10.1002/alz.12006

[28] Adhikari S , Qiao Y , Singer M , et al. Retinotopic degeneration of the retina and optic tracts in autosomal dominant Alzheimer's disease. Alzheimers Dement. 2023. doi:10.1002/alz.13100 PMC10603214 37102308

[29] Kashani AH , Asanad S , Chan JW , et al. Past, present and future role of retinal imaging in neurodegenerative disease. Prog Retin Eye Res. 2021;83:100938. 33460813 10.1016/j.preteyeres.2020.100938 PMC8280255

[30] Dumitrascu OM , Rosenberry R , Sherman DS , et al. Retinal venular tortuosity jointly with retinal amyloid burden correlates with verbal memory loss: a pilot study. Cells. 2021;10. 10.3390/cells10112926 PMC8616417 34831149

[31] Shi H , Koronyo Y , Rentsendorj A , et al. Identification of early pericyte loss and vascular amyloidosis in Alzheimer's disease retina. Acta Neuropathol. 2020;139:813‐836. 32043162 10.1007/s00401-020-02134-w PMC7181564

[32] Shi H , Koronyo Y , Fuchs DT , et al. Retinal arterial Abeta(40) deposition is linked with tight junction loss and cerebral amyloid angiopathy in MCI and AD patients. Alzheimers Dement. 2023. doi:10.1002/alz.13086 PMC10638467 37166032

[33] La Morgia C , Ross‐Cisneros FN , Koronyo Y , et al. Melanopsin retinal ganglion cell loss in Alzheimer disease. Ann Neurol. 2016;79:90‐109. 26505992 10.1002/ana.24548 PMC4737313

[34] Koronyo Y , Biggs D , Barron E , et al. Retinal amyloid pathology and proof‐of‐concept imaging trial in Alzheimer's disease. JCI Insight. 2017;2. 10.1172/jci.insight.93621 PMC5621887 28814675

[35] Shi H , Koronyo Y , Fuchs DT , et al. Retinal capillary degeneration and blood‐retinal barrier disruption in murine models of Alzheimer's disease. Acta Neuropathol Commun. 2020;8:202. 33228786 10.1186/s40478-020-01076-4 PMC7686701

[36] Shi H , Yin Z , Koronyo Y , et al. Regulating microglial miR‐155 transcriptional phenotype alleviates Alzheimer's‐induced retinal vasculopathy by limiting Clec7a/Galectin‐3(+) neurodegenerative microglia. Acta Neuropathol Commun. 2022;10:136. 36076283 10.1186/s40478-022-01439-z PMC9461176

[37] Singer MB , Ringman JM , Chu Z , et al. Abnormal retinal capillary blood flow in autosomal dominant Alzheimer's disease. Alzheimers Dement (Amst). 2021;13:e12162. 33728371 10.1002/dad2.12162 PMC7931411

[38] Ngolab J , Donohue M , Belsha A , et al. Feasibility study for detection of retinal amyloid in clinical trials: the anti‐amyloid treatment in asymptomatic Alzheimer's disease (A4) trial. Alzheimers Dement (Amst). 2021;13:e12199. 34430703 10.1002/dad2.12199 PMC8369843

[39] Tadokoro K , Yamashita T , Kimura S , et al. Retinal amyloid imaging for screening Alzheimer's disease. J Alzheimers Dis. 2021;83:927‐934. 34366344 10.3233/JAD-210327

[40] Hadoux X , Hui F , Lim JKH , et al. Non‐invasive in vivo hyperspectral imaging of the retina for potential biomarker use in Alzheimer's disease. Nat Commun. 2019;10:4227. 31530809 10.1038/s41467-019-12242-1 PMC6748929

[41] More SS , Beach JM , McClelland C , Mokhtarzadeh A , Vince R . In vivo assessment of retinal biomarkers by hyperspectral imaging: early detection of Alzheimer's disease. ACS Chem Neurosci. 2019;10:4492‐4501. 31603648 10.1021/acschemneuro.9b00331

[42] Lemmens S , Van Craenendonck T , Van Eijgen J , et al. Combination of snapshot hyperspectral retinal imaging and optical coherence tomography to identify Alzheimer's disease patients. Alzheimers Res Ther. 2020;12:144. 33172499 10.1186/s13195-020-00715-1 PMC7654576

[43] Koronyo‐Hamaoui M , Koronyo Y , Ljubimov AV , et al. Identification of amyloid plaques in retinas from Alzheimer's patients and noninvasive in vivo optical imaging of retinal plaques in a mouse model. Neuroimage. 2011;54(Suppl 1):S204‐S217.. 20550967 10.1016/j.neuroimage.2010.06.020 PMC2991559

[44] den Haan J , Morrema THJ , Verbraak FD , et al. Amyloid‐beta and phosphorylated tau in post‐mortem Alzheimer's disease retinas. Acta Neuropathol Commun. 2018;6:147. 30593285 10.1186/s40478-018-0650-x PMC6309096

[45] Grimaldi A , Pediconi N , Oieni F , et al. Neuroinflammatory processes, A1 astrocyte activation and protein aggregation in the retina of Alzheimer's disease patients, possible biomarkers for early diagnosis. Front Neurosci. 2019;13:925. 31551688 10.3389/fnins.2019.00925 PMC6737046

[46] Schultz N , Byman E , Netherlands Brain B , Wennstrom M . Levels of retinal amyloid‐beta correlate with levels of retinal IAPP and hippocampal amyloid‐beta in neuropathologically evaluated individuals. J Alzheimers Dis. 2020;73:1201‐1209. 31884473 10.3233/JAD-190868 PMC7081096

[47] Lee S , Jiang K , McIlmoyle B , et al. Amyloid beta immunoreactivity in the retinal ganglion cell layer of the Alzheimer's eye. Front Neurosci. 2020;14:758. 32848548 10.3389/fnins.2020.00758 PMC7412634

[48] Qiu Y , Jin T , Mason E , Campbell MCW . Predicting thioflavin fluorescence of retinal amyloid deposits associated with Alzheimer's disease from their polarimetric properties. Transl Vis Sci Technol. 2020;9:47. 10.1167/tvst.9.2.47 PMC7443113 32879757

[49] Xu QA , Boerkoel P , Hirsch‐Reinshagen V , et al. Muller cell degeneration and microglial dysfunction in the Alzheimer's retina. Acta Neuropathol Commun. 2022;10:145. 36199154 10.1186/s40478-022-01448-y PMC9533552

[50] Hart de Ruyter FJ , Morrema THJ , den Haan J , et al. Phosphorylated tau in the retina correlates with tau pathology in the brain in Alzheimer's disease and primary tauopathies. Acta Neuropathol. 2023;145:197‐218. 36480077 10.1007/s00401-022-02525-1

[51] Alexandrov PN , Pogue A , Bhattacharjee S , Lukiw WJ . Retinal amyloid peptides and complement factor H in transgenic models of Alzheimer's disease. Neuroreport. 2011;22:623‐627. 21734608 10.1097/WNR.0b013e3283497334 PMC3719862

[52] Tsai Y , Lu B , Ljubimov AV , et al. Ocular changes in TgF344‐AD rat model of Alzheimer's disease. Invest Ophthalmol Vis Sci. 2014;55:523‐534. 24398104 10.1167/iovs.13-12888 PMC3907137

[53] Cao KJ , Kim JH , Kroeger H , et al. ARCAM‐1 facilitates fluorescence detection of amyloid‐containing deposits in the retina. Transl Vis Sci Technol. 2021;10:5. 10.1167/tvst.10.7.5 PMC8185402 34096989

[54] Schon C , Hoffmann NA , Ochs SM , et al. Long‐term in vivo imaging of fibrillar tau in the retina of P301S transgenic mice. PLoS One. 2012;7:e53547. 23300938 10.1371/journal.pone.0053547 PMC3534024

[55] Nilson AN , English KC , Gerson JE , et al. Tau oligomers associate with inflammation in the brain and retina of tauopathy mice and in neurodegenerative diseases. J Alzheimers Dis. 2017;55:1083‐1099. 27716675 10.3233/JAD-160912 PMC5147514

[56] Ho CY , Troncoso JC , Knox D , Stark W , Eberhart CG . Beta‐amyloid, phospho‐tau and alpha‐synuclein deposits similar to those in the brain are not identified in the eyes of Alzheimer's and Parkinson's disease patients. Brain Pathol. 2014;24:25‐32. 23714377 10.1111/bpa.12070 PMC3976129

[57] Walkiewicz G , Ronisz A , Van Ginderdeuren R , et al. Primary retinal tauopathy: a tauopathy with a distinct molecular pattern. Alzheimers Dement. 2023. doi:10.1002/alz.13424 PMC10916964 37615275

[58] Du X , Koronyo Y , Mirzaei N , et al. Label‐free hyperspectral imaging and deep‐learning prediction of retinal amyloid beta‐protein and phosphorylated tau. PNAS Nexus. 2022;1:pgac164. 36157597 10.1093/pnasnexus/pgac164 PMC9491695

[59] Koronyo Y , Rentsendorj A , Mirzaei N , et al. Retinal pathological features and proteome signatures of Alzheimer's disease. Acta Neuropathol. 2023;145:409‐438. 36773106 10.1007/s00401-023-02548-2 PMC10020290

[60] Johnson ECB , Dammer EB , Duong DM , et al. Large‐scale proteomic analysis of Alzheimer's disease brain and cerebrospinal fluid reveals early changes in energy metabolism associated with microglia and astrocyte activation. Nat Med. 2020;26:769‐780. 32284590 10.1038/s41591-020-0815-6 PMC7405761

[61] Li X , Tsolis KC , Koper MJ , et al. Sequence of proteome profiles in preclinical and symptomatic Alzheimer's disease. Alzheimers Dement. 2021;17:946‐958. 33871169 10.1002/alz.12345

[62] Bambo MP , Garcia‐Martin E , Otin S , et al. Visual function and retinal nerve fibre layer degeneration in patients with Alzheimer disease: correlations with severity of dementia. Acta Ophthalmol. 2015;93:e507‐e508. 10.1111/aos.12635 25529560

[63] Hinton DR , Sadun AA , Blanks JC , Miller CA . Optic‐nerve degeneration in Alzheimer's disease. N Engl J Med. 1986;315:485‐487. 3736630 10.1056/NEJM198608213150804

[64] Dumitrascu OM , Lyden PD , Torbati T , et al. Sectoral segmentation of retinal amyloid imaging in subjects with cognitive decline. Alzheimers Dement (Amst). 2020;12:e12109. 33015311 10.1002/dad2.12109 PMC7521595

[65] Byun MS , Park SW , Lee JH , et al. Association of retinal changes with Alzheimer disease neuroimaging biomarkers in cognitively normal individuals. JAMA Ophthalmol. 2021;139:548‐556. 33764406 10.1001/jamaophthalmol.2021.0320 PMC7995126

[66] Asanad S , Fantini M , Sultan W , et al. Retinal nerve fiber layer thickness predicts CSF amyloid/tau before cognitive decline. PLoS One. 2020;15:e0232785. 32469871 10.1371/journal.pone.0232785 PMC7259639

[67] Salobrar‐Garcia E , de Hoz R , Ramirez AI , et al. Changes in visual function and retinal structure in the progression of Alzheimer's disease. PLoS One. 2019;14:e0220535. 31415594 10.1371/journal.pone.0220535 PMC6695171

[68] Kirbas S , Turkyilmaz K , Anlar O , Tufekci A , Durmus M . Retinal nerve fiber layer thickness in patients with Alzheimer disease. J Neuroophthalmol. 2013;33:58‐61. 22918296 10.1097/WNO.0b013e318267fd5f

[69] Kromer R , Serbecic N , Hausner L , Froelich L , Aboul‐Enein F , Beutelspacher SC . Detection of retinal nerve fiber layer defects in Alzheimer's disease using SD‐OCT. Front Psychiatry. 2014;5:22. 24616709 10.3389/fpsyt.2014.00022 PMC3934110

[70] Coppola G , Di Renzo A , Ziccardi L , et al. Optical coherence tomography in Alzheimer's disease: a meta‐analysis. PLoS One. 2015;10:e0134750. 26252902 10.1371/journal.pone.0134750 PMC4529274

[71] Santos CY , Johnson LN , Sinoff SE , Festa EK , Heindel WC , Snyder PJ . Change in retinal structural anatomy during the preclinical stage of Alzheimer's disease. Alzheimers Dement (Amst). 2018;10:196‐209. 29780864 10.1016/j.dadm.2018.01.003 PMC5956814

[72] Golzan SM , Goozee K , Georgevsky D , et al. Retinal vascular and structural changes are associated with amyloid burden in the elderly: ophthalmic biomarkers of preclinical Alzheimer's disease. Alzheimers Res Ther. 2017;9:13. 28253913 10.1186/s13195-017-0239-9 PMC5335799

[73] O'Bryhim BE , Apte RS , Kung N , Coble D , Van Stavern GP . Association of preclinical Alzheimer disease with optical coherence tomographic angiography findings. JAMA Ophthalmol. 2018;136:1242‐1248. 30352114 10.1001/jamaophthalmol.2018.3556 PMC6248182

[74] Doustar J , Rentsendorj A , Torbati T , et al. Parallels between retinal and brain pathology and response to immunotherapy in old, late‐stage Alzheimer's disease mouse models. Aging Cell. 2020;19:e13246. 33090673 10.1111/acel.13246 PMC7681044

[75] Chiasseu M , Alarcon‐Martinez L , Belforte N , et al. Tau accumulation in the retina promotes early neuronal dysfunction and precedes brain pathology in a mouse model of Alzheimer's disease. Mol Neurodegener. 2017;12:58. 28774322 10.1186/s13024-017-0199-3 PMC5543446

[76] Grimaldi A , Brighi C , Peruzzi G , et al. Inflammation, neurodegeneration and protein aggregation in the retina as ocular biomarkers for Alzheimer's disease in the 3xTg‐AD mouse model. Cell Death Dis. 2018;9:685. 29880901 10.1038/s41419-018-0740-5 PMC5992214

[77] Liu B , Rasool S , Yang Z , et al. Amyloid‐peptide vaccinations reduce beta‐amyloid plaques but exacerbate vascular deposition and inflammation in the retina of Alzheimer's transgenic mice. Am J Pathol. 2009;175:2099‐2110. 19834067 10.2353/ajpath.2009.090159 PMC2774073

[78] Ning A , Cui J , To E , Ashe KH , Matsubara J . Amyloid‐beta deposits lead to retinal degeneration in a mouse model of Alzheimer disease. Invest Ophthalmol Vis Sci. 2008;49:5136‐5143. 18566467 10.1167/iovs.08-1849 PMC3947384

[79] Perez SE , Lumayag S , Kovacs B , Mufson EJ , Xu S . Beta‐amyloid deposition and functional impairment in the retina of the APPswe/PS1DeltaE9 transgenic mouse model of Alzheimer's disease. Invest Ophthalmol Vis Sci. 2009;50:793‐800. 18791173 10.1167/iovs.08-2384 PMC3697019

[80] Zhang M , Zhong L , Han X , et al. Brain and retinal abnormalities in the 5xFAD mouse model of alzheimer's disease at early stages. Front Neurosci. 2021;15:681831. 34366774 10.3389/fnins.2021.681831 PMC8343228

[81] Rijal Upadhaya A , Kosterin I , Kumar S , et al. Biochemical stages of amyloid β‐peptide aggregation and accumulation in the human brain and their association with symptomatic and pathologically‐preclinical Alzheimer's disease. Brain. 2014;137:887‐903. 24519982 10.1093/brain/awt362

[82] Aragao Gomes L , Uytterhoeven V , Lopez‐Sanmartin D , et al. Maturation of neuronal AD‐tau pathology involves site‐specific phosphorylation of cytoplasmic and synaptic tau preceding conformational change and fibril formation. Acta Neuropathol. 2021;141:173‐192. 33427938 10.1007/s00401-020-02251-6

[83] Pediconi N , Gigante Y , Cama S , et al. Retinal fingerprints of ALS in patients: ganglion cell apoptosis and TDP‐43/p62 misplacement. Front Aging Neurosci. 2023;15:1110520. 37009460 10.3389/fnagi.2023.1110520 PMC10061015

[84] Doustar J , Torbati T , Black KL , Koronyo Y , Koronyo‐Hamaoui M . Optical coherence tomography in Alzheimer's disease and other neurodegenerative diseases. Front Neurol. 2017;8:701. 29312125 10.3389/fneur.2017.00701 PMC5742098

[85] Habiba U , Descallar J , Kreilaus F , et al. Detection of retinal and blood Abeta oligomers with nanobodies. Alzheimers Dement (Amst). 2021;13:e12193. 33977118 10.1002/dad2.12193 PMC8101010

[86] Hart NJ , Koronyo Y , Black KL , Koronyo‐Hamaoui M . Ocular indicators of Alzheimer's: exploring disease in the retina. Acta Neuropathol. 2016;132:767‐787. 27645291 10.1007/s00401-016-1613-6 PMC5106496

[87] Gupta N , Fong J , Ang LC , Yucel YH . Retinal tau pathology in human glaucomas. Can J Ophthalmol. 2008;43:53‐60. 18219347 10.3129/i07-185

[88] Xu L , Ryu J , Nguyen JV , et al. Evidence for accelerated tauopathy in the retina of transgenic P301S tau mice exposed to repetitive mild traumatic brain injury. Exp Neurol. 2015;273:168‐176. 26311071 10.1016/j.expneurol.2015.08.014

[89] Veys L , Vandenabeele M , Ortuno‐Lizaran I , et al. Retinal alpha‐synuclein deposits in Parkinson's disease patients and animal models. Acta Neuropathol. 2019;137:379‐395. 30721408 10.1007/s00401-018-01956-z

[90] Beach TG , Carew J , Serrano G , et al. Phosphorylated alpha‐synuclein‐immunoreactive retinal neuronal elements in Parkinson's disease subjects. Neurosci Lett. 2014;571:34‐38. 24785101 10.1016/j.neulet.2014.04.027 PMC4591751

[91] Ward ME , Taubes A , Chen R , et al. Early retinal neurodegeneration and impaired Ran‐mediated nuclear import of TDP‐43 in progranulin‐deficient FTLD. J Exp Med. 2014;211:1937‐1945. 25155018 10.1084/jem.20140214 PMC4172214

[92] Rijal Upadhaya A , Lungrin I , Yamaguchi H , Fändrich M , Thal DR . High‐molecular weight Aβ‐oligomers and protofibrils are the predominant Aβ‐species in the native soluble protein fraction of the AD brain. J Cell Mol Med. 2012;16:287‐295. 21418518 10.1111/j.1582-4934.2011.01306.x PMC3823292

[93] Ascaso FJ , Cruz N , Modrego PJ , et al. Retinal alterations in mild cognitive impairment and Alzheimer's disease: an optical coherence tomography study. J Neurol. 2014;261:1522‐1530. 24846203 10.1007/s00415-014-7374-z

[94] Jentsch S , Schweitzer D , Schmidtke KU , et al. Retinal fluorescence lifetime imaging ophthalmoscopy measures depend on the severity of Alzheimer's disease. Acta Ophthalmol. 2015;93:e241‐e247. 25482990 10.1111/aos.12609

[95] Einarsdottir AB , Hardarson SH , Kristjansdottir JV , Bragason DT , Snaedal J , Stefansson E . Retinal oximetry imaging in Alzheimer's disease. J Alzheimers Dis. 2016;49:79‐83. 26444785 10.3233/JAD-150457

[96] Koychev I , Lawson J , Chessell T , et al. Deep and frequent phenotyping study protocol: an observational study in prodromal Alzheimer's disease. BMJ Open. 2019;9:e024498. 10.1136/bmjopen-2018-024498 PMC6475176 30904851

[97] Csincsik L , Quinn N , Yong KXX , Crutch SJ , Peto T , Lengyel I . Retinal phenotyping of variants of Alzheimer's disease using ultra‐widefield retinal images. Alzheimers Dement (Amst). 2021;13:e12232. 34458553 10.1002/dad2.12232 PMC8377778

[98] Quinn N , Csincsik L , Flynn E , et al. The clinical relevance of visualising the peripheral retina. Prog Retin Eye Res. 2019;68:83‐109. 30316018 10.1016/j.preteyeres.2018.10.001

[99] Tewarie P , Balk L , Costello F , et al. The OSCAR‐IB consensus criteria for retinal OCT quality assessment. PLoS One. 2012;7:e34823. 22536333 10.1371/journal.pone.0034823 PMC3334941

[100] Veitch DP , Weiner MW , Aisen PS , et al. Using the Alzheimer's disease neuroimaging initiative to improve early detection, diagnosis, and treatment of Alzheimer's disease. Alzheimers Dement. 2022;18:824‐857. 34581485 10.1002/alz.12422 PMC9158456

[101] Vujosevic S , Cunha‐Vaz J , Figueira J , et al. Standardization of optical coherence tomography angiography imaging biomarkers in diabetic retinal disease. Ophthalmic Res. 2021;64:871‐887. 34348330 10.1159/000518620

[102] La Joie R , Ayakta N , Seeley WW , et al. Multisite study of the relationships between antemortem [(11)C]PIB‐PET Centiloid values and postmortem measures of Alzheimer's disease neuropathology. Alzheimers Dement. 2019;15:205‐216. 30347188 10.1016/j.jalz.2018.09.001 PMC6368897

[103] Cheung CY , Ran AR , Wang S , et al. A deep learning model for detection of Alzheimer's disease based on retinal photographs: a retrospective, multicentre case‐control study. Lancet Digit Health. 2022;4:e806‐e815. 36192349 10.1016/S2589-7500(22)00169-8

[104] Wisely CE , Wang D , Henao R , et al. Convolutional neural network to identify symptomatic Alzheimer's disease using multimodal retinal imaging. Br J Ophthalmol. 2022;106:388‐395. 33243829 10.1136/bjophthalmol-2020-317659

[105] Alafuzoff I , Pikkarainen M , Al‐Sarraj S , et al. Interlaboratory comparison of assessments of Alzheimer disease‐related lesions: a study of the BrainNet Europe Consortium. J Neuropathol Exp Neurol. 2006;65:740‐757. 16896308 10.1097/01.jnen.0000229986.17548.27

[106] Alafuzoff I , Pikkarainen M , Arzberger T , et al. Inter‐laboratory comparison of neuropathological assessments of beta‐amyloid protein: a study of the BrainNet Europe consortium. Acta Neuropathol. 2008;115:533‐546. 18343933 10.1007/s00401-008-0358-2

[107] Gattoussi S , Buitendijk GHS , Peto T , et al. The European eye epidemiology spectral‐domain optical coherence tomography classification of macular diseases for epidemiological studies. Acta Ophthalmol. 2019;97:364‐371. 30242982 10.1111/aos.13883

[108] Schneider JA , Arvanitakis Z , Bang W , Bennett DA . Mixed brain pathologies account for most dementia cases in community‐dwelling older persons. Neurology. 2007;69:2197‐2204. 17568013 10.1212/01.wnl.0000271090.28148.24

[109] Rahimi J , Kovacs GG . Prevalence of mixed pathologies in the aging brain. Alzheimers Res Ther. 2014;6:82. 25419243 10.1186/s13195-014-0082-1 PMC4239398

[110] Braak H , Del Tredici K . Alzheimer's pathogenesis: is there neuron‐to‐neuron propagation? Acta Neuropathol. 2011;121:589‐595. 21516512 10.1007/s00401-011-0825-z

[111] Brettschneider J , Del Tredici K , Lee VM , Trojanowski JQ . Spreading of pathology in neurodegenerative diseases: a focus on human studies. Nat Rev Neurosci. 2015;16:109‐120. 25588378 10.1038/nrn3887 PMC4312418

[112] Ye L , Hamaguchi T , Fritschi SK , et al. Progression of seed‐induced abeta deposition within the limbic connectome. Brain Pathol. 2015;25:743‐752. 25677332 10.1111/bpa.12252 PMC4530099

[113] Thal DR , Rub U , Orantes M , Braak H . Phases of A beta‐deposition in the human brain and its relevance for the development of AD. Neurology. 2002;58:1791‐1800. 12084879 10.1212/wnl.58.12.1791

[114] Braak H , Braak E . Neuropathological stageing of Alzheimer‐related changes. Acta Neuropathol. 1991;82:239‐259. 1759558 10.1007/BF00308809

